# Kidney Pathology Precedes and Predicts the Pathological Cascade of Cerebrovascular Lesions in Stroke Prone Rats

**DOI:** 10.1371/journal.pone.0026287

**Published:** 2011-10-21

**Authors:** Stefanie Schreiber, Celine Z. Bueche, Cornelia Garz, Siegfried Kropf, Doerthe Kuester, Kerstin Amann, Hans-Jochen Heinze, Michael Goertler, Klaus G. Reymann, Holger Braun

**Affiliations:** 1 Department of Neurology, Otto-von-Guericke University, Magdeburg, Germany; 2 Institute of Biometry and Medical Informatics, Otto-von-Guericke University, Magdeburg, Germany; 3 Institute of Pathology, Otto-von-Guericke University, Magdeburg, Germany; 4 Department of Nephropathology, University of Erlangen, Erlangen, Germany; 5 Deutsches Zentrum für Neurodegenerative Erkrankungen, Magdeburg, Germany; 6 Leibniz Institute for Neurobiology, Magdeburg, Germany; Julius-Maximilians-Universität Würzburg, Germany

## Abstract

**Introduction:**

Human cerebral small vessel disease (CSVD) has been hypothesized to be an age-dependent disease accompanied by similar vascular changes in other organs. SHRSP feature numerous vascular risk factors and may be a valid model of some aspects of human CSVD. Here we compare renal histopathological changes with the brain pathology of spontaneously hypertensive stroke-prone rats (SHRSP).

**Material and Methods:**

We histologically investigated the brains and kidneys of 61 SHRSP at different stages of age (12 to 44 weeks). The brain pathology (aggregated erythrocytes in capillaries and arterioles, microbleeds, microthromboses) and the kidney pathology (aggregated erythrocytes within peritubular capillaries, tubular protein cylinders, glomerulosclerosis) were quantified separately. The prediction of the brain pathology by the kidney pathology was assessed by creating ROC-curves integrating the degree of kidney pathology and age of SHRSP.

**Results:**

Both, brain and kidney pathology, show an age-dependency and proceed in definite stages whereas an aggregation of erythrocytes in capillaries and arterioles, we parsimoniously interpreted as stases, represent the initial finding in both organs. Thus, early renal tubulointerstitial damage characterized by rather few intravasal erythrocyte aggregations and tubular protein cylinders predicts the initial step of SHRSPs' cerebral vascular pathology marked by accumulated erythrocytes. The combined increase of intravasal erythrocyte aggregations and protein cylinders accompanied by glomerulosclerosis and thrombotic renal microangiopathy in kidneys of older SHRSP predicts the final stages of SHRSPs' cerebrovascular lesions marked by microbleeds and thrombotic infarcts.

**Conclusion:**

Our results illustrate a close association between structural brain and kidney pathology and support the concept of small vessel disease to be an age-dependent systemic pathology. Further, an improved joined nephrologic and neurologic diagnostic may help to identify patients with CSVD at an early stage.

## Introduction

Human cerebral small vessel disease (CSVD) is one of the major causes for both, stroke-like symptoms due to lacunar infarctions [1], and, cognitive decline leading to (vascular) dementia in a growing older population [2]. Associated histological changes of human CSVD mainly affect the arterioles and include blood brain barrier (BBB) disturbances indicated by plasma protein leakage into the vessel wall, concentric hyaline vessel wall thickening with subsequent stenosis, vessel occlusions, microaneurysms and hemorrhagic extravasation throughout the arteriolar wall leading to microbleeds [3]–[5].

Interestingly, in numerous human studies a simultaneous occurrence of CSVD and an impaired function of other organs including the kidney (decrease of glomerular filtration rate, proteinuria [6], [7]) and the retina [8] has been claimed. This observation suggests that the small vessel disease (SVD) exhibits a systemic condition leading to a common “arteriolar dysfunction” resulting in similar histological abnormalities of the “vascular beds” in different organs [9]. Age is therefore the major independent risk factor for the occurrence of small vessel changes; additional vascular risk factors accelerate the development of the SVD in multiple organs, of which arterial hypertension is the most important risk factor for brain SVD [9].

Because of its in vivo accessibility the structural microvascular changes of the human retina, including retinal microaneurysms, hemorrhages, arteriovenous nicking and focal arteriolar narrowing, are well established in patients with CSVD [8]. Contrary, although there is broad clinical literature concerning the association between a decline in renal function and CSVD [6], [10], the accompanying structural kidney changes are not well understood.

Thus, we aimed to describe the histological similarities and associations between cerebral and renal (vascular) changes found at different stages of age in male spontaneously hypertensive stroke-prone rats (SHRSP) exhibiting different vascular risk factors including arterial hypertension, insulin resistance and mixed hyperlipidemia. SHRSP may be a valid model of some aspects of human CSVD [11], [12].

Our results demonstrate that an accumulation of erythrocytes in capillary segments represent the quite homogeneous initial step of the renal and cerebral pathology in SHRSP. Both, cerebral and kidney pathology, proceed in definite age-dependent stages significantly associated between both organs. Thereby the kidney pathology precedes the brain pathology and may function as a predictor of brain pathology.

## Materials and Methods

### Animals

Animal procedures were conducted after obtaining the approval of the Animal Care Committee of Sachsen Anhalt (reference number of licence for animal testing 42502-2-943, July 2009, Magdeburg, Sachsen-Anhalt). Animals were housed with a natural light-dark cycle and allowed to access water and food *ad libitum*.

Sixty four male SHRSP were examined; one rat died during catheterization and two died spontaneously for reasons that remain unclear. Therefore, sixty one SHRSP (Charles River Laboratories International, Inc., Wilmington, MA, USA) were investigated histologically (12 weeks n = 6, 14 weeks n = 5, 16 weeks n = 3, 18 weeks n = 3, 20 to 26 weeks n = 4, 28 weeks n = 8, 30 to 32 weeks n = 10, 34 to 36 weeks n = 9, 39 to 42 weeks n = 10, 44 weeks n = 3). They were monitored daily for alterations in neurological status and body weight. Sixteen Wistar rats at corresponding ages served as control group (12 weeks n = 2, 18 weeks n = 4, 26 weeks n =  3, 32 weeks n = 2, 36 weeks n = 3, 65 weeks n = 2).

### Histology

#### Brain

Rats were transcardially perfused with 120 ml PBS in order to remove the blood completely, followed by 120 ml 4% PFA within 4 min. Brains and kidneys were removed, stored in 4% PFA for 48 hours, placed for cryoprotection into 30% sacharose for 6 days, and frozen in methylbutane at −80°C.

Coronal slices (30 µm) of the whole brain were prepared with a cryotoma (Leica, Nussloch, Germany) and stained with hematoxylin/eosin (HE) (for detail see [13]).

All brains of SHRSP and controls were investigated for the occurrence of vessels with accumulated erythrocytes, the occurrence of microbleeds and microthromboses [13].

#### Kidney

Fourteen µm slices of the kidney were prepared with a cryotoma (Leica, Nussloch, Germany) and stained with HE. From the cranial pole to the mid of the kidney there were 3 to 4 sectional planes (distance 1 mm), for histological investigation there were 9 to 12 slices per animal.

**Figure 1:**
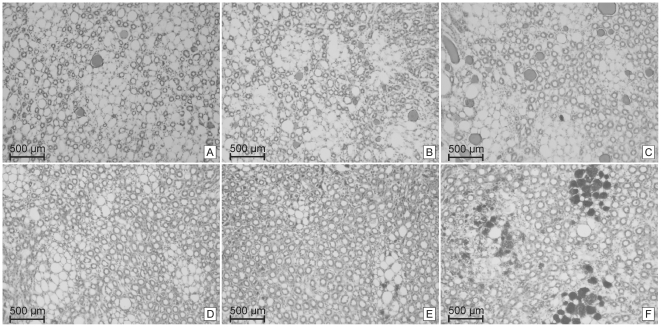
Quantification of tubular protein cylinders and of erythrocyte aggregations within peritubular capillaries. Kidney medulla. **A**: tubular protein cylinders in <5% per field of view ( = 1), **B**: tubular protein cylinders in >5% per field of view ( = 2), **C**: tubular protein cylinders in >30% per field of view ( = 3), **D**: peritubular aggregated erythrocytes in <5% per field of view ( = 1), **E**: peritubular aggregated erythrocytes in >5% per field of view ( = 2), **F**: peritubular aggregated erythrocytes in >30% per field of view ( = 3).

For all SHRSP and controls we separately assessed the occurrence of aggregated erythrocytes within peritubular capillaries and the occurrence of tubular protein cylinders in 6 HE slices per animal at all stages of age in a semiquantitative manner: aggregated erythrocytes or tubular protein cylinders per field of view (FOV) 0 = none, 1 =  <5%, 2 =  >5%, 3 =  >30% (*Figure 1*). We investigated 5 FOV (magnification 150x) per slice for kidney cortex and medulla each.

The occurrence of segmental and global glomerulosclerosis was assessed in the renal cortex.

#### Urine

Every 2^nd^ week we investigated the spontaneous morning urine (Combur, ROCHE DIAGNOSTICS GMBH, test strips) in 32 SHRSP aged 28 to 44 weeks and in 12 controls aged 12 to 36 weeks. We assessed glucose (0 = negative [normal], 1≤50 mg/dl, 2≤100 mg/dl, 3≥100 mg/dl), erythrocytes (0 = negative, 1 = 5–10/µl, 2≤50/µl, 3≥50/µl), protein (0 = negative, 1≤30 mg/dl, 2≤100 mg/dl, 3≥100 mg/dl), leukocytes (0 = negative, 1≤75/µl, 2≤500/µl, 3≥500/µl).

### Serum Glucose

In 29 SHRSP aged 28 weeks or older and in 6 controls aged 26 weeks or older the serum glucose (CONTOUR, Bayer, test strips) was assessed transcardially immediately before perfusion. Serum glucose is given in mmol/l.

### Statistics

#### Brain

The occurrence of erythrocyte accumulations in capillaries and arterioles (existent or not existent) at all stages of age was evaluated in the whole brain of all SHRSP and all controls. The occurrence of microbleeds (existent or not existent) and microthromboses (existent or not existent) at all stages of age was analyzed in the whole brain of all SHRSP (control animals did not exhibit any microbleeds or microthromboses). Using the two-sided linear-by-linear association chi-square test (exact version) the occurrence of erythrocyte accumulations, microbleeds and microthromboses at all stages of age was compared across the age groups of the SHRSP and the controls [13].

#### Kidney

To compare SHRSP and controls and to assess a possible age-dependency of kidney pathology we used a univariate analysis of variance with group (SHRSP, controls) as fixed factor and age as covariate. Thus while proving an age-dependency of kidney pathology in both groups we performed an age corrected group comparison. The results were presented by interpretation of the regression coefficient for age (including the 95% confidence interval and the p-value for the test against zero) and by the results of the F test for the group effect.

Using Spearman's and Pearson's correlation coefficient and logistic regression analysis we assessed the association between kidney pathology, single urine data, serum glucose and age in SHRSP. After proving an age-dependency of kidney pathology and urine parameters we performed a partial correlation with age as controlled factor. The results of that partial correlation and logistic regression analysis were taken for data interpretation.

The association between the quantified brain pathology (including the occurrence of erythrocyte aggregations in capillaries and arterioles, microbleeds, microthromboses) and the quantified kidney pathology (including the occurrence of erythrocyte aggregations within peritubular capillaries, tubular protein cylinders, glomerulosclerosis) in SHRSP was tested by a Mann-Whitney-U test/ROC-analysis without correction for age and by logistic regression analysis/ROC-analysis with correction for age. The results are presented in Table 1 as area under the curve (AUC), the 95% confidence interval and the p-value.

**Table 1:** Association between the quantified brain and kidney pathology.

Histopathology brain	Histopathology kidney	SHRSP							
		Mann-Whitney-U test without correction for age				Logistic regression analysis with correction for age			
		AUC	95% confidence intervall		p-value	AUC	95% confidence intervall		p-value
Erythrocyte aggregations	Erythrocyte aggregations medulla	0.789	0.590	0.989	**0.003**	0.863	0.769	0.957	0.294
	Erythrocyte aggregations cortex	0.764	0.558	0.970	**0.007**	0.844	0.731	0.958	0.689
	Protein cylinders medulla	0.796	0.630	0.962	**0.002**	0.864	0.763	0.964	0.382
	Protein cylinders cortex	0.769	0.588	0.949	**0.005**	0.861	0.755	0.967	0.290
	Glomerulosclerosis	0.753	0.590	0.916	**0.004**	0.850	0.737	0.963	0.760
Microbleeds	Erythrocyte aggregations medulla	0.862	0.773	0.952	**<0.001**	0.885	0.802	0.968	**0.029**
	Erythrocyte aggregations cortex	0.783	0.611	0.865	**0.003**	0.877	0.791	0.964	0.209
	Protein cylinders medulla	0.815	0.710	0.919	**<0.001**	0.873	0.788	0.959	0.831
	Protein cylinders cortex	0.745	0.622	0.868	**0.002**	0.867	0.779	0.955	0.821
	Glomerulosclerosis	0.767	0.653	0.882	**<0.001**	0.877	0.791	0.962	0.998
Microthromboses	Erythrocyte aggregations medulla	0.901	0.821	0.982	**0.004**	0.895	0.815	0.974	0.074
	Erythrocyte aggregations cortex	0.774	0.625	0.923	**0.067**	0.816	0.712	0.920	0.165
	Protein cylinders medulla	0.991	0.000	1.000	**<0.001**	0.991	0.000	1.000	0.103
	Protein cylinders cortex	0.978	0.000	1.000	**<0.001**	0.982	0.000	1.000	**0.035**
	Glomerulosclerosis	0.702	0.509	0.894	**0.158**	0.768	0.607	0.928	0.998

The statistical association between the quantified brain pathology (erythrocyte aggregations, microbleeds, microthromboses) and the quantified kidney pathology (erythrocyte aggregations, tubular protein cylinders, glomerulosclerosis) in SHRSP is demonstrated. Note the significant association between almost all cerebral and renal histopathological phenomena by using the Mann-Whitney-U test/ROC-analysis. After correction for age (logistic regression analysis/ROC-analysis) a significant association only remained between the occurrence of cerebral microbleeds and erythrocyte aggregations in the renal medulla as well as between the occurrence of cerebral mircothromboses and protein cylinders in the kidney cortex. AUC  =  the area under the curve.

Sensitivity and specificity for the prediction of brain pathology by kidney pathology were assessed by creating ROC-curves integrating the degree of kidney pathology and age of SHRSP (*Table 2*).

**Table 2:** The kidney pathology predicts the brain pathology in SHRSP.

	Erythrocyte accumulations brain	Microbleeds brain	Microthrom-boses brain
Erythrocyte aggregations kidney cortex	0.04/FOV (Se 88.2%, Sp 70%)	0.55/FOV (Se 83.3%, Sp 61.5%)	0.92/FOV (Se 100%, Sp 61.4%)
Erythrocyte aggregations kidney medulla	0.17/FOV (Se 86.3%, Sp 80%)	1.18/FOV (Se 77.8%, Sp 83.7%)	2.18/FOV (Se 100%, Sp 82.5%)
Protein cylinders kidney cortex	0.02/FOV (Se 78.4%, Sp 80%)	0.19/FOV (Se 100%, Sp 56.8%)	1.89/FOV (Se 100%, Sp 96.5%)
Protein cylinders kidney medulla	0.14/FOV (Se 80.4%, Sp 80%)	1.45/FOV (Se 83.3%, Sp 67.4%)	2.85/FOV (Se 100%, Sp 98.2%)

Table 2 demonstrates the sensitivity (Se) and specificity (Sp) of the graduation of kidney pathology (*rows*, kidney pathology per field of view (FOV), see also *Figure 1* and Material & Methods) to predict brain pathology (*columns*) in SHRSP. Data were assessed by creating ROC-curves integrating the degree of the kidney pathology and the age of SHRSP. Kidney pathology with the highest combined sensitivity and specificity predicts certain stages of brain pathology. Note that the advanced stages of the brain pathology are associated with a more severe graduation of the kidney pathology.

P-values less than 0.05 were deemed to be statistically significant.

## Results

None of the animals suffered from obvious neurological symptoms. One rat died during catheterization and two died spontaneously for reasons that remain unclear.

### Brain

**Figure 2:**
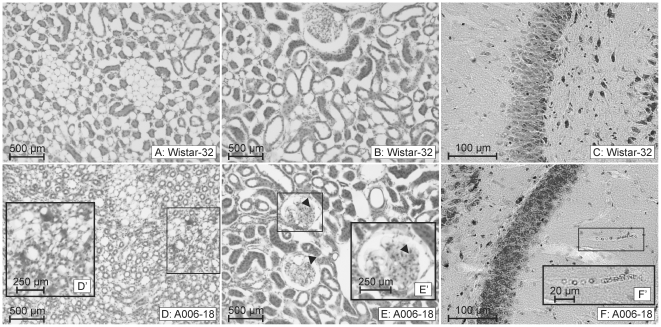
Kidney and brain morphology of a control and SHRSP with few cerebral erythrocyte accumulations. Both the kidney medulla (**A**) and cortex (**B**) of a 32 weeks old Wistar rat are free of peritubular erythrocyte aggregations, tubular protein cylinders, glomerulosclerosis and thrombotic microangiopathy which corresponds to normal brain morphology (here hippocampus, **C**). In contrast the kidney of an 18 weeks old SHRSP (animal 6) shows a few erythrocyte aggregations in medullar peritubular capillaries (**D** **&** **D'**) and erythrocyte sticking (*black arrow heads in* **E** **&** **E'**) in few glomeruli (**E** **&** **E'**). This pathology corresponds to mild erythrocyte accumulations (beginning stases) within some capillaries of the brain (here hippocampus, **F** **&** **F'**). Wistar-32: wistar control, 32 weeks old; A006-18: animal number 6, 18 weeks old.

**Figure 3:**
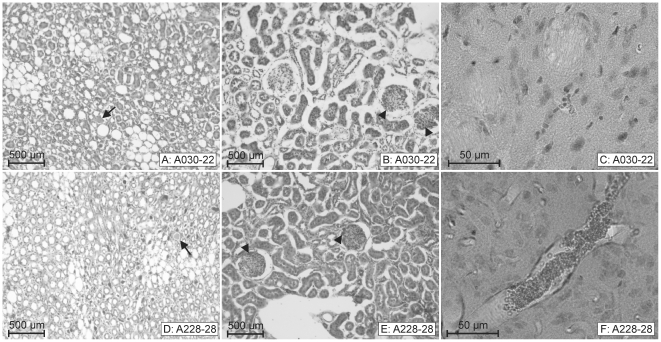
Two SHRSP with comparable peritubular erythrocyte aggregations, but a different extent of cerebral erythrocyte accumulations. **A–C**: SHRSP aged 22 weeks (animal 30). **D–F**: SHRSP aged 28 weeks (animal 228). Both animals exhibit comparable peritubular erythrocyte aggregations in kidney medulla (visible as red dots in **A** **&** **D**) as well as comparable glomerular erythrocyte sticking in kidney cortex (*black arrow heads in* **B** **&** **E**). The medulla of both kidneys additionally contains few tubular protein cylinders (*black arrows in* **A** **&** **D**). In contrast to the similar kidney pathology animal 30 exhibits in the brain only a marginal number of capillaries with accumulated erythrocytes (here basalganglia, **C**), whereas in animal 228 those accumulated erythrocytes are already found in cortical arterioles (**F**) and in arterioles of other brain regions (not shown). A030-22: animal number 30, 22 weeks old.

In a former study we demonstrated the proceeding of the cerebral vascular pathology in SHRSP in definite stages [13]. Thereby an accumulation of erythrocytes in the small vessels of the whole brain seemed to represent the homogeneous initial step of the disease (*Figure 2 F &* *Figure 3 C, F*). We referred to these accumulated erythrocytes parsimoniously as stases. The number of SHRSP with accumulated erythrocytes significantly increased with age. In SHRSP aged 28 weeks and older erythrocyte accumulations occurred in both, capillaries and arteriolar segments (*Figure 3 F*), whereas the accumulated erythrocytes found in younger animals (12 to 26 weeks) were seen predominantly in capillaries (*Figure 2 F &* *Figure 3 C*). About 60% of the SHRSP aged 12 weeks exhibited erythrocyte accumulations. Interestingly, at the age of 28 weeks we found accumulated erythrocytes in all investigated SHRSP (*Table S1*).

Those intravasal erythrocyte accumulations we occasionally also detected in the capillaries of the controls, but with a significant lower frequency and extent compared to the SHRSP.

**Figure 4:**
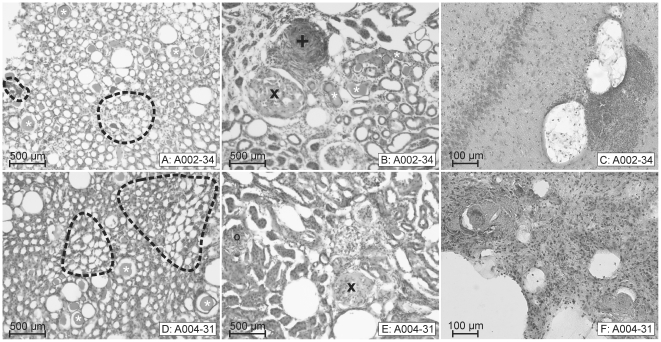
Severe kidney pathology in SHRSP corresponds to final stages of cerebral vascular pathology. **A–C**: SHRSP (animal 2, 34 weeks old) with cerebral microbleeds. **D–F**: SHRSP (animal 4, 31 weeks old) with cerebral infarctions containing small vessel occlusions. There is a strong correlation between an advanced kidney pathology, i.e. peritubular aggregations of erythrocytes, protein cylinders and different stages of glomerulosclerosis with cerebral infarctions accompanied by either microbleeds (here hippocampus, **C**) or small vessel occlusions with surrounding necrotic tissue (**F**). **A** **&** **D**: kidney medulla, **B** **&** **E**: kidney cortex, peritubular aggregations of erythrocytes are marked by *black dashed lines* (**A** **&** **D**), protein cylinders by *white asterisks* (**A**, **B** **&** **D**). Different stages of glomerulosclerosis are marked in **B** **& E** (***o***, ***x***; see also *Figure 5*). In addition kidney vessels are affected by thrombotic microangiopathy (***+*** *in* **B**). **B** corresponds to *Figure 5* **E**. A002-34: animal number 2, 34 weeks old.

When the SHRSP reached an age of about 32 weeks we detected extraluminal fresh microbleeds characterized by numerous grouped erythrocytes leaking from the small vessels into the parenchyma (*Figure 4 C*). Microthromboses (*Figure 4 F*) with subsequent cerebral infarctions characterized by spongy and cystic tissue destruction represent the final step in the pathological cascade of the cerebral vascular pathology in SHRSP.

**Figure 5:**
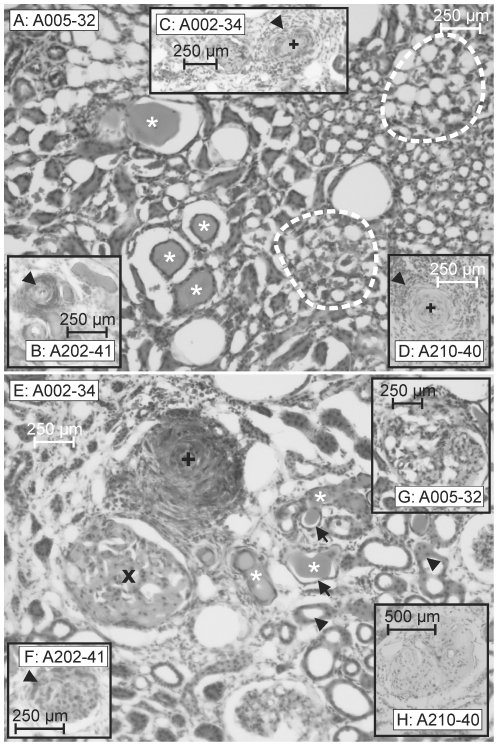
Structural damage of the kidney medulla and kidney cortex in SHRSP. *Figure 5* demonstrates the main histopathological changes in the kidney of SHRSP. **A** shows an area of dilated peritubular capillaries with intravasal erythrocyte aggregations in the kidney medulla (*marked by white dotted lines*). Note tubular protein cylinders (*white asterisks*) in the kidney medulla (**A**) and kidney cortex (**E**) indicating tubular damage. In addition, tubular damage is characterized by flattening and atrophy of the tubular epithelial cells (*black arrows*, **E**). Note in contrast the normal cubic epithelium of non damaged tubules (*black arrow heads*, **E**). In **B–E** chronological stages of thrombotic microangiopathy are illustrated. **B** **&** **E** demonstrate early, **C** **&** **D** demonstrate advanced stages. In **B** **&** **E** occluded arterioles with concentric thickened and hemorrhagic tunica media (“onion-skin”; *black arrow head in* **B**; *black cross in* **E**) are shown. **C** demonstrates a fresh microthrombosis (*black cross*) with excentric hemorrhage and **D** shows an older microthrombosis (*black cross*). Note the accompanying leukocyte leakage into the perivascular interstitium (*black arrow heads*, **C** **&** **D**). In **E–H** chronological stages of glomerulosclerosis are illustrated. **F** demonstrates an early stage marked by erythrocyte sticking in glomerular capillaries *(black arrow head*); in **E**, **G** **&** **H** advanced stages with capillary obliteration and increased mesangial matrix (*black x*, **E**) leading to a segmental (**G**) or more global (**H**) glomerulosclerosis. A005-32: animal number 5, 32 weeks old.

None of the controls exhibited microbleeds, microthromboses and tissue infarctions.

### Kidney

Kidneys from SHRSP exhibited a variety of pathological changes much stronger expressed in older animals (*Table S1*). Kidney pathology includes erythrocyte aggregations, protein cylinders, glomerulosclerosis and thrombotic microangiopathy.

#### Peritubular dilated capillaries with intravasal erythrocyte aggregations

We detected aggregated erythrocytes within the peritubular capillaries of kidney cortex and medulla (*Figure 5 A*). Most of those capillaries seemed to be dilated. The peritubular intravasal erythrocyte aggregations closely resembled those erythrocyte accumulations we detected in capillaries and arterioles of the brain. Therefore, similar to the brain, we referred to the renal erythrocyte aggregations parsimoniously as stases. In the kidney medulla we found most of those aggregated erythrocytes, probably caused by the medullas higher blood supply compared to the kidney cortex.

**Figure 6:**
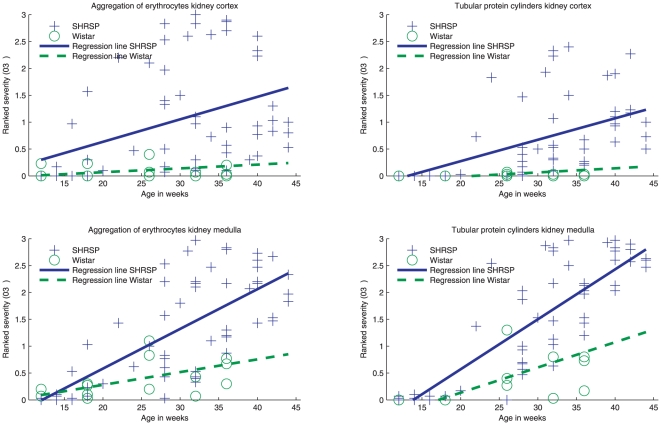
Association between the kidney pathology and age in SHRSP and Wistar rats. Regression lines. The regression lines in *Figure 6* demonstrate the positive association between the severity of the kidney pathology (y-axis) and the age (x-axis) in SHRSP (blue) and Wistar (green) group. The extent of peritubular aggregated erythrocytes and the extent of tubular protein cylinders is separately assessed in the kidney medulla and cortex and is captured in a semiquantitative manner (0–3, y-axis; see also *Figure 1* *and* Material and Methods, *kidney*). Note the increase of the severity of the kidney pathology with age in SHRSP (*cortex*: erythrocyte aggregations, regression coefficient [rc] 0.042, CI 0.019-0.065, p = 0.001; protein cylinders, rc 0.04, CI 0.025–0.055, p = <0.001; *medulla*: erythrocyte aggregations, rc 0.074, CI 0.057–0.090, p = <0.001; protein cylinders, rc 0.093, CI 0.079–0.107, p = <0.001) and to a lesser extent in the Wistar group (*cortex*: erythrocyte aggregations, rc 0.07, CI -0.001–0.015, p = 0.08; protein cylinders, rc 0.008, CI 0.005–0.011, p = <0.001; *medulla*: erythrocyte aggregations, rc 0.024, CI 0.014–0.034, p = <0.001; protein cylinders, rc 0.047, CI 0.033–0.061, p = <0.001) resulting in significant group differences (*cortex*: erythrocyte aggregations p = 0.045, protein cylinders p = 0.004; *medulla*: erythrocyte aggregations p = <0.001, protein cylinders p = <0.001).

Single erythrocytes seemed to migrate throughout the vessel wall into the tubulointerstitial tissue; a process we also detected in the brain and we referred to as diapedesis. Aggregated erythrocytes were detected in kidneys of SHRSP and controls. However, in controls those intravasal erythrocyte aggregations occurred with a significant lower frequency (*Figure 6*). In both, SHRSP and controls, there was an age-dependent increase of the erythrocyte aggregations in kidney cortex and medulla (*Figure 6*).

#### Protein cylinders

Secondly, we detected tubular protein cylinders in kidney cortex and medulla (*Figure 5* *A*, *E*). Protein cylinders indicate a thickened tubular secretion (Tamm-Horsfall glycoprotein) depositing as a consequence of acute and chronic tubular damage. Furthermore, tubular damage was marked by a flattening and an atrophy of the tubular epithelial cells (*Figure 5 E*).

Moreover, we found different intensities of the protein cylinders ‘staining (*Figure 5 E*) indicating the duration of the tubular damage. A higher staining intensity of a protein cylinder displayed a higher concentrated secret correlating to a longer lasting tubular damage.

Tubular protein cylinders occurred in SHRSP and controls. However, in controls we detected protein cylinders with a significant lower frequency (*Figure 6*). In both, SHRSP and controls, there was an age-dependent increase of protein cylinders in kidney cortex and medulla (*Figure 6*).

#### Glomerulosclerosis

Glomerulosclerosis was characterized by obliteration of glomerular capillaries, mesangial matrix deposition and mesangial cell proliferation (*Figure 5 E–H*). Additionally, in early stages of glomerulosclerosis there were numerous sticking erythrocytes and leukocytes localized in the glomerular capillaries and the mesangial matrix (*Figure 3 B, E &* *Figure 5 F*). The final stage of glomerulosclerosis was characterized by marked obliteration of the capillary lumina; in general we found only few or no sticking erythrocytes and leukocytes at that stage (*Figure 5 H*). Moreover, we detected both a segmental glomerulosclerosis, affecting only a portion of the glomerular tuft (<50%) and a global glomerulosclerosis, affecting most of the glomerular tuft (*Figure 5 E, G, H*).

Occasionally we detected intravasal erythrocyte aggregations in the glomerular afferent and efferent arterioles. Those erythrocyte aggregations closely resembled those ones we found in the peritubular capillaries and the small brain vessels.

In SHRSP we found a significant age-dependency of glomerulosclerosis. However, none of the controls exhibited a glomerulosclerosis.

#### Thrombotic Microangiopathy (TMA)

In several small vessels of kidney cortex and medulla we found a so-called TMA characterized by concentric vessel wall thickening (“onion-skin”) and microthromboses (*Figure 5 B–E*). Microthromboses and vessel occlusions were often accompanied by leukocytes leaking into the perivascular interstitium (*Figure 5 C, D*).

None of the controls exhibited a TMA.

### Urine and blood glucose

All SHRSP aged 28 weeks and nearly all controls aged 26 weeks exhibited a proteinuria of at least ≤100 mg/dl. 90.6% (29 of 32) SHRSP had a hematuria of at least 5–10 erythrocytes/µl. Only one of 12 controls (8.3%) exhibited a microhematuria ( = 1). Starting at an age of 28 weeks, in 13 of 32 SHRSP (40.6%) we detected a glucosuria, significantly increasing with age (Spearman's correlation coefficient 0.442, p = 0.011; Pearson's correlation coefficient 0.435, p = 0.013). In 9 of 13 SHRSP with glucosuria (69.2%) the blood glucose reached normal values. None of the controls exhibited a glucosuria.

There were no statistical associations between kidney pathology and single urine parameters and between kidney pathology and serum glucose.

### Association between kidney and brain pathology illustrated in exemplary SHRSP

As already outlined in our first study [13] SHRSP develop an age-dependent cascade of their cerebral vascular pathology beginning with accumulations of erythrocytes in small vessels, followed by microbleeds which are finally converted into reactive vessel occlusions. Microbleeds and microthromboses are consequently found to be dispersed in infarcted regions marked by necrotic tissue. Similar to the brain the kidney pathology seemed to develop along a certain cascade.

Thereby, as an initial step of kidney pathology we detected peritubular erythrocyte aggregations, immediately followed or even paralleled by tubular protein cylinders (*Table S1*). Those initial changes were often accompanied by sticking of erythrocytes throughout the glomerular capillary endothelial cells (*Figure 3 B, E*). Both, an increasing extent of erythrocyte aggregations or protein cylinders, and, an increasing combined occurrence of erythrocyte aggregations and protein cylinders, seemed to indicate more severe kidney pathology (*Table S1*). Segmental, global glomerulosclerosis and TMA were common features of final kidney damage. Moreover, the severity of the kidney pathology was associated with the severity of the brain pathology (*Table 2*).

Interestingly, in all three SHRSP, illustrated in *Figure 2* *and* *Figure 3*, exhibiting the initial step of the cerebral vascular pathology we found features characteristic for early to less severe steps of the kidney damage (peritubular erythrocyte aggregations accompanied by single protein cylinders and glomerular erythrocyte sticking, *Figure 2 D, E*, *Figure 3 A, B, D, E*). *Figure 3* also demonstrates that initial and comparable kidney damage between two SHRSP may be associated with different stages of development of erythrocyte accumulations in the brain. Thus, animal 30 (*Figure 3 A–C*) has only few accumulated erythrocytes in brain capillaries, whereas animal 228 (*Figure 3 D–F*) has frequent and big erythrocyte accumulations even in brain arterioles, although the kidney pathology of both is nearly the same. This suggests that kidney pathology develops before cerebral small vessel disease, which is also supported by the fact, that in SHRSP with cerebral microbleeds (animal 2, 34 weeks, *Figure 4 C*), microthromboses and subsequent tissue infarctions (animal 4, 31 weeks, *Figure 4* F), representing the final stages of cerebral vascular pathology, we regularly detected a severe and final kidney pathology. Final kidney pathology comprehends extended peritubular erythrocyte aggregations combined with extended tubular protein cylinders (*Figure 4 A, D*), TMA (*Figure 4 B*), segmental and global glomerulosclerosis (*Figure 4 B, E*).

### Statistical association between kidney and brain pathology in SHRSP

All stages of brain pathology [13] and all quantified features of kidney pathology showed a significant age-dependency (*Figure 6*).

The association between quantified features of brain pathology and quantified features of kidney pathology are illustrated in Table 1.

Further, taken the severity of kidney pathology and the age of SHRSP into consideration, there was a high combined sensitivity and specificity to predict the single stages of the brain pathology by knowing the severity of the kidney pathology (*Table 2*).

Thus, a combined increase of peritubular erythrocyte aggregations (0.92 kidney cortex; 2.18 kidney medulla per; 150x per FOV) and tubular protein cylinders (1.89 kidney cortex; 2.85 kidney medulla; 150x per FOV) correlated with 100% sensitivity for microthrombotic brain infarctions in the same SHRSP (*Table 2*).

Further, statistical analysis suggested that pathologies in kidney medulla have a higher correlation to brain pathology than structural changes in kidney cortex.

## Discussion

Human CSVD affects small arteries, arterioles and capillaries [3], [4]. However, despite several common risk factors including age, arterial hypertension, diabetes mellitus and hyperlipidemia knowing to accelerate human CSVD, the initial step and the details of the progression of that disease are not clarified [9].

Recently we demonstrated that the cerebral vascular pathology in SHRSP proceeds in definite temporal stages whereas an accumulation of erythrocytes in the whole brain capillaries and arterioles represent the initial step of the disease. BBB disturbances, microbleeds and reactive microthromboses leading to necrotic tissue infarctions with fresh and old hemorrhages represent the subsequent stages of the pathological cerebral vascular cascade [13].

Interestingly, there are hints in the literature that human CSVD is part of a systemic dysfunction of the small arteries and arterioles (“vascular bed”) affecting multiple organs [9]. However, it is not well understood whether the different organs are affected simultaneously or sequentially by the damage of the “vascular bed”.

We therefore investigated the association between the histopathological changes in the brain and the kidneys of SHRSP. Interestingly, thereby we depicted a definite chronological development of the vascular kidney pathology comparable to that one we were able to demonstrate in the brain. Kidney damage was described partially in former SHRSP studies [14] and resembles those histopathological changes found in hypertensive nephropathy [15].

Our results demonstrate that erythrocyte aggregations in peritubular capillaries (*Figure 2 D*) represent the initial step of the kidney damage. According to the erythrocyte accumulations we detected in the small brain vessels [13], we parsimoniously interpret the renal erythrocyte aggregations as stases. Additional histological changes we depicted as part of an early structural kidney pathology include hyaline tubular protein cylinders (“pseudothyroid areas”, [15]; *Figure 5* E), erythrocytes aggregating within the glomerular afferent and efferent arterioles and accumulated erythrocytes within the mesangial matrix (“erythrocyte sticking”) (*Figure 2 E, E'*).

Peritubular erythrocyte aggregations and tubular protein cylinders are part of tubulointerstitial kidney damage. This tubulointerstitial pathology is the consequence but also the cause of an arterial hypertension [16].

Those erythrocytes aggregating in the glomerular afferent arterioles might be associated with a reduced blood flow velocity leading to an impairment of glomerular perfusion pressure and to glomerular hypoperfusion [17]. A subsequent transient glomerular ischemia results in an increase of the permeability of the capillary endothelium. That increased permeability might be causal for the glomerular erythrocyte and leukocyte “sticking” we detected in several SHRSP (*Figure 2 E, E'*). Moreover, glomerular hypoperfusion results in an increased release of renin, leading to deterioration of arterial hypertension [18]. To regulate against that vicious circle, SHRSP aged 6 weeks and older exhibit a “myogenic response” resulting in an increased vascular constriction and smaller afferent arteriolar diameters [19].

The histological phenomena of the early structural kidney damage occur separately or combined in kidney medulla (*Figure 1 &* *Figure 5 A*) and cortex (*Figure 3 B, E &* *Figure 5 E*). Interestingly, proven by both, a qualitative analysis of histology (*Figure 2 &* *Figure 3*) and a quantitative evaluation of the brain and kidney pathology (*Table 1*, *Table 2*), there is a correlation between the early kidney damage and the initial step of the cerebral vascular pathology (erythrocyte accumulations) in SHRSP.

The progression of kidney damage is marked by an increase of the tubulointerstitial damage. Moreover, the combined increase of peritubular erythrocyte aggregations and tubular protein cylinders is accompanied by different stages of glomerulosclerosis and TMA (*Figure 4* *&* *Figure 5*).

As one of the main conclusions of our study we depicted a high association between this extended combined tubulointerstitial kidney pathology and an advanced cerebral vascular pathology including microbleeds and thrombotic tissue infarctions (*Figure 4* *&* *Table 2*).

Additionally, similar to an age-dependent cerebral vascular pathology, we recently demonstrated in SHRSP [13], logistic regression analysis also revealed an age-dependency of the kidney pathology in SHRSP and controls (*Figure 6*). Thus, our results support the hypothesis of microangiopathy to be an age-dependent systemic condition, deteriorated by vascular risk factors [9] and kidney pathology obviously precedes the brain pathology.

By the means of exemplary animals (*Figure 2 &* *Figure 3*) we demonstrate, that the kidney damage occurs first and remains stable during a certain but undefined time period. In contrast, the cerebral vascular pathology seems to be the consequence of renal changes and progresses over a period, in which kidney pathology holds steady.

As already mentioned, erythrocytes, accumulating in small vessels, represent the initial step of the histopathological cascade in both kidney and brain. Thereby, the small renal and brain vessels (arterioles) fulfill autoregulatory properties to keep a constant perfusion pressure of the adjacent tissue [20]. Thus, the aggregating erythrocytes we found in the arterioles of brain and kidney, we parsimoniously interpret as stases, seem to be the consequence of autoregulatory disturbances, associated reductions of the blood flow velocity and possible vessel wall changes.

Erythrocyte accumulations represent the quite homogeneous initial step of the cerebral vascular pathology in SHRSP. This early stage is not accompanied by a destruction of the adjacent brain tissue including infarctions and necrosis and, remains unchanged over several weeks [13]. Contrary, those early peritubular capillary erythrocyte aggregations we found in the kidney are rapidly associated with further kidney damage: The erythrocyte aggregations lead to hypoperfusion with a subsequent hypoxic-ischemic damage of the adjacent tubular epithelial cells [15], [21], characterized by an epithelial atrophy and a thickened tubular secretion (*Figure 5 E*). The depositing protein cylinders obstruct the tubular lumen and therefore deteriorate the tubular damage [22]. Longer lasting tubulointerstitial kidney damage is marked by a higher staining intensity of a protein cylinder displaying a higher concentrated secret (*Figure 4 B*).

Erythrocytes and leukocytes accumulating within the mesangial matrix indicate an early glomerular damage (*Figure 5 F*); moreover, those aggregating cells induce the development of glomerulosclerosis marked by an obliteration of glomerular capillaries and mesangial cell proliferation [15] in its advanced stages (*Figure 4 B, E &* *Figure 5 H*). Segmental glomerulosclerosis precedes global glomerulosclerosis [15], which is exemplary illustrated by animal 210 concurrently possessing extended cerebral infarctions (*Figure 5 H*).

An extended tubulointerstitial damage including both, peritubular erythrocyte aggregations and tubular protein cylinders, was generally associated with TMA [15], [17] (*Figure 5 B–E*). TMA is commonly accompanied by perivascular inflammation [15], [23] (*Figure 5 C, D*). In those SHRP exhibiting renal TMA we regularly detected cerebral hemorrhages and brain infarctions with microthromboses, additionally supporting the correlation between an advanced kidney and brain pathology.

Concluding, the kidney pathology in SHRSP including tubulointerstitial damage, glomerulosclerosis and TMA associated with interstitial inflammation resemble those pathological changes found in hypertensive nephropathy [15]. Thus, in SHRSP renal damage seems to be caused by an arterial hypertension, obviously evoked by disturbed interactions between prostaglandins and the adrenergic nerve system [24], genetic dysregulation of the renin-angiotensin system, occurrence of vascular wall renin [25] and elevated plasma renin activity [26].

As already shown in former investigations [14] we detected proteinuria of comparable extent in SHRSP and controls aged about 28 weeks. Older SHRSP, aged at least 45 weeks, exhibit an urinary protein excretion (albuminuria) significantly exceeding the urinary protein values measured in controls [14]. The proteinuria in young animals, we found in both groups, seems to be associated with tubulointerstitial damage [22] (*Figure 6*). However, there was no statistical association between proteinuria and the extent of tubulointerstitial damage. Contrary, the proteinuria detected in older SHRSP is the consequence of a disturbed filtration caused by glomerulosclerosis [14] being part of an advanced kidney pathology.

In several SHRSP aged 28 weeks and older we also detected an age-dependent glucosuria accompanied by normal values of the serum glucose. Consequently, in most of the SHRSP glucosuria seems to be a further consequence of the age-related structural kidney damage rather than of a diabetes mellitus.

One limitation of our study is the missing determination of further parameters, including creatinine clearance, glomerular filtration rate and plasma creatinine concentration, indicating renal function. However, those data have been adequately investigated by Feld et al., who were able to show, that those parameters are affected for the first time in SHRSP aged 50 weeks and older [14].

In conclusion, we found significant associations between the SHRSPs' renal and cerebral vascular pathology. Both, kidney and brain damage, show a significant age-dependency.

An early kidney damage indicated by a little extent of tubulointerstitial damage including peritubular erythrocyte aggregations and protein cylinders accompanied by glomerular erythrocyte “sticking”, predicts the initial step of the cerebral vascular pathology marked by accumulated erythrocytes within the small brain vessels. Interestingly, even some of the older controls exhibited the same association between the early kidney and brain pathology. Contrary, in SHRSP with extended tubulointerstitial damage accompanied by glomerulosclerosis and TMA, we detected an advanced cerebral vascular pathology characterized by hemorrhages, microthromboses and associated tissue infarctions.

Our results support the hypothesis of microangiopathy to be an age-dependent systemic condition [9]. Since kidney pathology obviously precedes brain pathology, an improved joined nephrologic and neurologic diagnostics may thereby lead to an early identification of patients with high risks for CSVD.

## 

## Supporting Information

Table S1 **Severity of the renal and cerebral histopathologies at different ages in SHRSP and Wistar rats.** The severity of the cerebral (including erythrocyte aggregations, microbleeds, microthromboses) and renal (including erythrocyte aggregations, tubular protein cylinders, glomerulosclerosis) histopathologies in all SHRSP and control animals is illustrated for every single animal. For the kidney the severity of aggregated erythrocytes and tubular protein cylinders (0 = none, 1 =  <5%, 2 =  >5%, 3  =  >30%, see also *Figure 1* and Material and Methods) was assessed in 5 fields of view (FOV) per slice in 6 HE slices per animal. In this table the mean values of all examined 30 FOV per animal are given. The associated pathologies, including glomerulosclerosis in the kidney and erythrocyte aggregations, microbleeds, or microthromboses in the brain were assessed in a binary manner: 0  =  not existent, 1  =  existent. The severity of renal erythrocyte aggregations and protein cylinders is visualized by different shades of red; see the scale at the bottom of the table. Note the increasing severity of the different histopathologies with age in the SHRSP and the control group. However, in controls the different histopathologies occurred with a lower frequency and severity (see also *Figure 6*). Note that the cerebral and renal pathological cascade started with erythrocyte aggregations. In the kidney, those accumulated erythrocytes progressively extended and were partially accompanied by protein cylinders. Renal glomerulosclerosis, cerebral microbleeds and microthromboses represented the final stages of the kidney and brain pathologies in SHRSP.
(PDF)
