# Supplementary material for: Kidney Pathology Precedes and Predicts the Pathological Cascade of Cerebrovascular Lesions in Stroke Prone Rats
Source: PLoS One. 2011 Oct 21;6(10):e26287. doi: 10.1371/journal.pone.0026287 (PMC3198774; doi:10.1371/journal.pone.0026287)
Supplement: Table S1 — Severity of the renal and cerebral histopathologies at different ages in SHRSP and Wistar rats. The severity of the cerebral (including erythrocyte aggregations, microbleeds, microthromboses) and renal (including erythrocyte aggregations, tubular protein cylinders, glomerulosclerosis) histopathologies in all SHRSP and control animals is illustrated for every single animal. For the kidney the severity of aggregated erythrocytes and tubular protein cylinders (0 = none, 1 = <5%, 2 = >5%, 3 = >30%, see also Figure 1 and Material and Methods) was assessed in 5 fields of view (FOV) per slice in 6 HE slices per animal. In this table the mean values of all examined 30 FOV per animal are given. The associated pathologies, including glomerulosclerosis in the kidney and erythrocyte aggregations, microbleeds, or microthromboses in the brain were assessed in a binary manner: 0 = not existent, 1 = existent. The severity of renal erythrocyte aggregations and protein cylinders is visualized by different shades of red; see the scale at the bottom of the table. Note the increasing severity of the different histopathologies with age in the SHRSP and the control group. However, in controls the different histopathologies occurred with a lower frequency and severity (see also Figure 6 ). Note that the cerebral and renal pathological cascade started with erythrocyte aggregations. In the kidney, those accumulated erythrocytes progressively extended and were partially accompanied by protein cylinders. Renal glomerulosclerosis, cerebral microbleeds and microthromboses represented the final stages of the kidney and brain pathologies in SHRSP. (PDF) [file pone.0026287.s001.pdf]

Age in weeks	Kidney					Brain		
	Cortex Bleeds	Proteins	Marrow Bleeds	Proteins	Glomerulo-sklerose	Stases	Microbleeds	Thromboses
SHRSP								
12	0.00	0.00	0.03	0.03	0	1	0	0
	0.00	0.00	0.03	0.07	0	1	0	0
	0.00	0.00	0.00	0.00	0	0	0	0
	0.00	0.00	0.00	0.00	0	1	0	0
	0.00	0.00	0.00	0.00	0	0	0	0
	0.00	0.00	0.00	0.00	0	1	0	0
14	0.17	0.00	0.07	0.03	0	0	0	0
	0.00	0.00	0.00	0.00	0	1	0	0
	0.00	0.00	0.10	0.10	0	0	0	0
	0.00	0.00	0.00	0.00	0	0	0	0
	0.00	0.00	0.00	0.00	0	0	0	0
16	0.97	0.00	0.53	0.00	0	1	0	0
	0.00	0.00	0.03	0.07	0	0	0	0
	0.00	0.00	0.00	0.00	0	0	0	0
18	0.00	0.00	0.07	0.07	0	1	0	0
	1.57	0.00	1.03	0.07	0	1	0	0
	0.30	0.00	0.23	0.07	0	1	0	0
20 - 26	0.10	0.03	0.30	0.17	0	1	0	0
	2.20	0.73	1.43	1.37	1	1	0	0
	0.47	1.83	0.62	2.54	1	1	0	0
	2.10	0.00	1.00	0.00	1	1	0	0
28	0.17	1.47	0.43	2.03	1	1	0	0
	1.33	0.27	0.60	0.70	1	1	0	0
	1.40	0.07	0.83	0.67	1	1	0	0
	2.53	0.17	2.20	0.93	1	1	0	0
	0.50	0.00	0.77	0.47	0	1	0	0
	0.07	0.53	0.03	1.87	1	1	0	0
	2.83	0.03	2.53	0.57	1	1	0	0
	1.97	0.40	1.57	1.00	1	1	0	0
30 - 32	1.50	0.50	1.80	1.53	1	0	0	0
	2.60	1.93	2.77	2.87	1	1	1	1
	3.00	2.23	2.97	2.77	1	0	1	0
	0.97	2.33	2.10	2.83	1	1	1	0
	1.10	0.33	2.17	1.47	1	1	1	0
	0.13	1.07	0.33	2.17	0	1	0	0
	0.30	0.10	0.43	1.13	0	1	0	0
	0.13	0.53	0.53	1.03	0	1	0	0
	0.10	0.57	0.40	1.13	0	1	0	0
	2.83	0.30	2.20	0.63	1	1	1	0
34 - 36	2.63	2.40	2.47	2.97	1	1	0	1
	0.73	1.50	1.10	2.50	1	1	0	0
	2.87	0.23	2.80	1.20	1	1	1	0
	0.10	0.20	0.87	1.43	1	1	1	0
	0.90	0.27	1.20	2.03	1	1	1	0
	0.43	0.50	1.17	1.97	1	1	0	0
	2.70	0.67	2.17	2.13	1	1	1	0
	2.90	0.23	2.83	1.53	1	1	1	0
	0.57	0.27	1.30	2.17	1	1	1	0
39 - 42	0.30	1.87	2.07	2.93	1	1	0	0
	2.60	1.10	2.60	2.77	1	1	0	0
	0.77	1.20	2.17	2.13	1	1	1	0
	2.33	0.97	2.73	2.60	1	1	0	0
	2.23	0.63	2.47	2.03	1	1	0	0
	0.93	1.90	2.20	2.97	1	1	1	1
	0.37	0.93	1.43	2.83	1	1	1	0
	1.03	1.23	1.53	2.57	1	1	0	0
	0.83	1.17	1.47	2.80	1	1	0	0
	1.30	2.27	2.67	2.90	1	1	1	1
44	0.80	1.00	2.33	2.47	1	1	1	0
	1.00	0.73	1.83	2.60	1	1	1	0
	0.53	0.50	1.97	2.63	1	1	1	0
Wistar								
12	0.00	0.00	0.07	0.00	0	0	0	0
	0.23	0.00	0.20	0.00	0	1	0	0
18	0.00	0.00	0.10	0.00	0	0	0	0
	0.23	0.00	0.27	0.00	0	0	0	0
	0.00	0.00	0.03	0.00	0	0	0	0
	0.00	0.00	0.30	0.00	0	0	0	0
26	0.07	0.07	0.83	0.27	0	1	0	0
	0.00	0.00	0.20	0.40	0	0	0	0
	0.40	0.03	1.10	1.30	1	0	0	0
32	0.00	0.00	0.43	0.03	0	0	0	0
	0.07	0.03	0.07	0.80	0	1	0	0
36	0.00	0.03	0.30	0.73	0	1	0	0
	0.03	0.00	0.77	0.80	0	0	0	0
	0.20	0.00	0.67	0.17	0	1	0	0
65	0.00	0.57	1.57	2.90	0	0	0	0
	0.97	0.27	1.27	2.07	0	0	0	0

Severity	0	0.01 - 0.49	0.50 - 0.99	1.00 - 1.49	1.50 - 1.99	2.00 - 2.49	2.50 - 3.00
